# On the “Strength” of Behavior

**DOI:** 10.1007/s40614-020-00269-5

**Published:** 2020-11-10

**Authors:** Carsta Simon, João Lucas Bernardy, Sarah Cowie

**Affiliations:** 1 University of Agder, Postboks 422, 4604 Kristiansand, Norway; 2 University of São Paulo, São Paulo, Brazil; 3 University of Auckland, Auckland, New Zealand

**Keywords:** Response strength, Signposts, Strengthening by reinforcement, Discrete units, Response reservoir, Private events, Molar approach

## Abstract

The place of the concept of response strength in a natural science of behavior has been the subject of much debate. This article reconsiders the concept of response strength for reasons linked to the foundations of a natural science of behavior. The notion of response strength is implicit in many radical behaviorists’ work. Palmer (2009) makes it explicit by applying the response strength concept to three levels: (1) overt behavior, (2) covert behavior, and (3) latent or potential behavior. We argue that the concept of response strength is superfluous in general, and an explication of the notion of giving causal status to nonobservable events like latent behavior or response strength is harmful to a scientific endeavor. Interpreting EEG recordings as indicators of changes in response strength runs the risk of reducing behavior to underlying mechanisms, regardless of whether such suggestions are accompanied by behavioral observations. Many radical behaviorists understand behavior as a discrete unit, inviting conceptual mistakes reflected in the notion of response strength. A molar view is suggested as an alternative that accounts for the temporally extended nature of behavior and avoids the perils of a response-strength based approach.

The science of behavior focuses on functional relations among behavior and the stimulating environment. As a natural science there is a focus on observable happenings. Although this focus may seem simple enough at first glance, studying functional relations among behavior and the stimulating environment involves a number of nuanced complexities. One such complexity pertains to the alleged *strength* of a behavior, whereby some behaviors are assumed to be strong (e.g., a loud vocalization, a forceful jump, or highly efficient lever presses in the animal lab) and others weak (e.g., briefly thinking about something, smiling slightly, speaking with uncertainty). The notion that behavior might have *strength* is not new; behavior analysts have discussed the topic of response strength for many years (de Villiers & Herrnstein, 1976; Herrnstein, 1961; Meehl, 1950; Nevin, 1974). Skinner (1938, p. 15) defines response strength as encompassing all static properties of the response. Measures proposed as indications of a response’s strength are the rate, latency, and resistance to extinction (e.g., Killeen & Hall, 2001), as well as the response’s force, prepotency over assumed competing responses, and related neural activity, such as that measured by electroencephalographic (EEG) activity (e.g., Palmer, 2009). Skinner (1938, 1953, 1974) discussed strength in some of his most influential writings. The notion of *strength* is today implicit in many behavior analytic theories (e.g., Nevin & Grace, 2000) and treatments (e.g., Cowdery, Iwata, & Pace, 1990). A hallmark of response-strength–based accounts is that they treat behavior as discretized units.

The history of the currently omnipresent concept of *strength* began with the Associationists or Connectionists, who thought of the strength of a bond between one thought or movement and another (Timberlake, 1988). This origin shows itself in Skinner’s (1948) superstition paper and his following work. When bond strength was then tied to frequency, contiguity, and effect, strength seemed to have a referent in observable features of the environment. Following Thorndike, Skinner started with reflexes and S-R bond, but when he conceived of operant behavior in the absence of an antecedent stimulus, response strength became troublesome. First, Skinner tried to equate response strength with the number of responses made in extinction, but when this proved an unreliable measure, he continued to write of response strength with no clear referent. Since then, the field has made various attempts to rescue the concept of strength (e.g., Nevin & Grace, 2000; Palmer, 2009; see Meehl, 1950; Postman, 1947; Shahan, 2017, for detailed discussion) with relatively limited success (e.g., see Craig & Shahan, 2016). Nevertheless, response-strength approaches to understanding behavior remain at the heart of much theory and application.

In this article, in line with other recent commentary (e.g., Cowie, 2019; Cowie & Davison, 2016; Killeen & Jacobs, 2017; Shahan, 2010, 2017; Simon, 2020), we argue that the notion of *response strength* brings us no closer to understanding behavior from a natural science perspective because it is unclear what *response strength* applies to, or how it should be measured. Does response strength apply only to overt behavior? If so, is *response strength* absolute frequency, relative frequency, resistance to disruption, or stimulus control? If yelling is considered strong and whispering weak, what about whispering to the person next to you for the entire duration of a lecture, versus yelling out the answer to the lecturer’s question so others can hear you just once in the course of your entire 4-year degree? Does the relative strength of these two responses reverse, depending on the timescale on which they are measured? Does response strength also apply to latent behavior? If so, does it apply to neurobiological or private events, and what are these? The concept of *response strength* may generate questions, but its ambiguity is a major barrier to providing definitive answers.

Indeed, behavior analysts’ struggle with response strength is highlighted by a recent discussion of the topic by prominent researchers in behavior analysis (e.g., Killeen & Jacobs, 2017; Shahan, 2017). The field is divided in its opinion on the place of response strength in a science of behavior. On the one hand, behavior analysts have criticized the notion of response strength (e.g., Baum, 2012; Bolles, 1975; Davison & Baum, 2006; Longstreth, 1971; Rachlin, 1976; Shahan, 2010; Simon, 2020; Staddon, 1983, 2016; Timberlake, 1988), largely as a result of its failure to predict or explain behavior. Other behavior analysts regard strength as a necessary component. For example, Palmer (2009) proposes that the notion of “response strength [does] more good than harm” (p. 59). It is clear that there is need for discussion about the place of response strength in a science of behavior.

Palmer (2009) suggests a way of overcoming the ambiguity considering both measurement and definition of response strength. This conceptualization of response strength attempts to account for control of behavior by multiple stimuli, and how organisms handle time gaps between stimuli and responses. Palmer’s conceptualization argues for response strength as a useful part of the explanation of behavior. Having grown out of the discussion about limits to a simple conceptualization of response strength, Palmer’s conceptualization is today likely to make explicit the mainstream radical behavior analysts’ implicit views on response strength. Hence, in the following, we discuss the perils of the concept of response strength per se, using Palmer’s approach in particular because it is both one of the rare explications of implicit mainstream views of response strength (Shahan, 2017) and an example of the adverse consequences of adopting a response-strength–based approach. We first consider Palmer’s conceptualization of *strengthening* by reinforcement in detail, and how it might be used to bridge time gaps between explanatory variables by proposing hypothetical latent events. The hazards of such an attempt are considered, including why calling alleged latent events “behavioral” is not a workable resolution. Finally, we consider a molar^1^ approach (Baum, 2013, 2016, 2018) as an alternative account of behavior without response strength, and without the need to discretize behavior.

## Palmer’s Conceptualization of Response Strength

Palmer (2009) suggests that an explanation of everyday behavior in complex environments requires the concept of response strength. He argues that the hypothetical construct of response strength is useful in predicting future behavior, discussing the status of absent behavior, and interpreting seemingly elementary cognitive phenomena such as problem solving and recall. It is traditional to say a reinforcer strengthens a response or stimulus–response connection when the reinforcer occurs contiguously with a response (Skinner, 1948; Thorndike, 1911/2000 ). Changes in response strength are thus assumed to occur as a consequence of a response being followed by a reinforcer. As Timberlake (1988, p. 311) summarizes Skinner’s view:

learning consists of the strengthening of responding. . . . The strengthening metaphor lived on in the focus on response-reward proximity and frequency. . . . The influence of the strengthening assumption is nowhere clearer than in Skinner's (1948) analysis of superstitious behavior in the pigeon. In this procedure pigeons were presented food periodically in a response independent fashion (a fixed-time schedule), and some movement typically increased in rate. Skinner attributed this result to reinforcement based on the assumption of differential temporal contiguity between the food and the movement. In this use, reinforcement was not related to any production operations, but was inferred solely from an outcome and attributed to an unsubstantiated proximity relation between food and an unspecified response candidate. This use of reinforcement seems uncomfortably similar to the invoking of mentalistic causes that Skinner himself argued against so well.

**Fig. 1:**
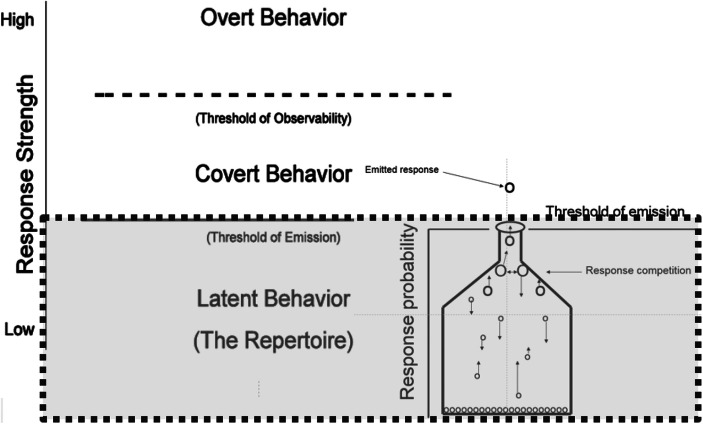
Arrangement of palmer’s (2009) figures 1 and 2 showing the location of the bottleneck metaphor in the domain of latent responses

In this contiguity-based approach, the response and an event that is assumed to function as a reinforcer are observed, and because the response (given the presence of the relevant discriminative stimuli) occurs more frequently in the future, we talk about a larger response probability or response strength. This simple concept is difficult to apply to situations where behavior comes under control of one or more stimuli that are temporally distant from the behavior (e.g., Cowie, Davison, & Elliffe, 2017). Palmer (2009) suggests expanding the application of the notion of response strength across three “levels of behavior” (see Figure 1) to address various problems, including the effect of the sequential onset of multiple stimuli on an organism’s behavior.

The first level consists of overt behavior, that is, behavior observed by an external observer. The second level consists of covert behavior, that is, events inferred from an introspector’s verbal behavior (e.g., “I’m thinking about going to the gym”). The third level consists of latent behavior, that is, events which have not occurred whatsoever (i.e., behavior that is not occurring either overtly [level 1] or covertly [level 2]). Note that a change in response strength can occur on the third level without postulating that the response has been or will ever be emitted (see Palmer, 2009, p. 53).

Figure 1 shows a combination of Palmer’s (2009) Figures 1 and 2 in which the three levels of behavior are depicted on the left. The proposed events on the third—latent—behavioral level, in the gray box, are represented by his bottleneck metaphor on the right. Circles on the bottom of the flask represent responses in an organism’s behavioral repertoire. This repertoire has strong parallels to the idea of an operant reserve, replacing the original idea of a reflex reserve (cf. Catania, 2005, 2017; Killeen, 1988; Timberlake, 1988; Skinner, 1940). Responses in the repertoire are neither necessarily observed, nor emitted; they are only potential behavior. If, owing to a history of exposure to contingencies of reinforcement, an organism’s response has come under control of various independent variables, it becomes part of that organism’s repertoire. Palmer postulates a latent competition of responses in the repertoire, the winner of which raises from the latent behavior level to the overt or covert level, and is, thus, the emitted response. The y-axis shows response strength, increasing from latent to covert to overt responses and corresponding to response probability on the latent level. Note that overt responses are not claimed to necessarily have a higher response strength than covert or latent responses, as the arrangement in the figure might imply. Whether or not a response with a certain strength is emitted depends rather on the strength of the competing latent responses. The y-axis is not supposed to imply that all responses are necessarily first latent and then covert before becoming overt (Palmer, personal communication, July 2, 2016).

**Fig. 2:**
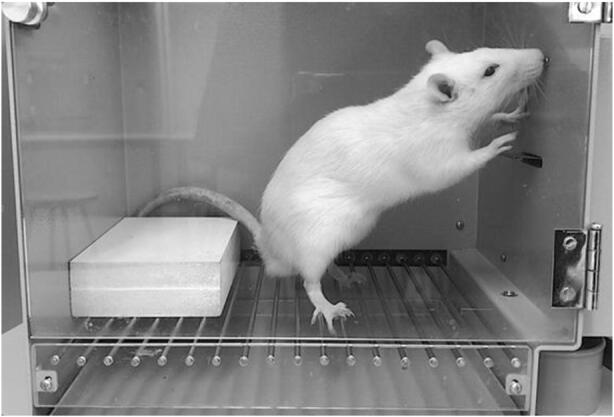
A snapshot that is not enough to be sure what the rat is doing

With growing response strength responses move to the emission threshold. When a response is suddenly emitted, Palmer (2009) “believe[s] that the reason is that in many contexts, the relevant responses already exist just below threshold strength. A small increase in strength is sufficient for the response to be emitted” (p. 57). One-trial learning may be explained as a result of this same process.

Palmer (2009) endorses and elaborates on the need for the concept of response strength by suggesting that the concept has both practical value (i.e., in the prediction of behavior) and interpretative value (i.e., conceptualizing behavior that is not observed). To Palmer, a response can have “a much higher strength even though, in a sense, the response does not yet exist” (p. 50). Behavior that is private or not yet observed is the source of much debate and controversy in behavior analysis. Indeed, although including such behavior seems to broaden the scope of our interpretive efforts, at the same time it threatens the fundamental assumption that behavior is amenable to confrontation in the world of nature. If we assume a cause of behavior to be unobservable or private by definition, we fall into a circularity. The only alternative for radical behaviorists is to state that privacy is a practical problem, and “private events” may be observed as soon as proper equipment is developed (Baum, 2011a). If there were a machine that could print one’s thoughts after measuring physiological activity, how could we assess the correspondence between the physiological measure and the “private behavior” printed on the paper? If the participant denied that they were thinking what the machine said they were, how could we decide if the participant was lying? (see Baum, 2011a, for a discussion). Assuming such a machine is possible implies neural identity theory, that states that specific behavioral topographies are reliably related to patterns of brain activity (see Rachlin, 2012, 2014; Skinner, 1938).

Arguments for and against including private behavior in a science of behavior have been well-articulated over the years (e.g., Baum, 2011a, 2011b, 2011c; Hayes & Fryling, 2009; Marr, 2011a, 2011b; Moore, 2009; Palmer, 2011; Rachlin, 2011), and we do not intend to revisit them in detail. Rather, our goal is to address the concept of response strength, where it results from a readiness to include private events in the analysis of behavior.

## Applying Response Strength to Account for Behavioral Phenomena

To Palmer (2009) the concept of response strength is, for example, useful for explaining the notion of multiple control as exemplified by the sequential occurrence of two stimuli leading to a different or faster occurring response than the presentation of one of the stimuli alone. Priming studies, for instance, show faster reactions to “robin” when the word “bird” was shown before. In word association games, the answer “Darwin” might not be given when hearing “famous male” alone but might occur when also hearing “natural selection.” Thus, reading “bird” changes the response strength of “robin,” and reading “famous male” and “natural selection” both have cumulative effects on the response strength of “Darwin.”

The response strength notion applied to latent “behavior” allows the time gap between stimulus and response occurrence to be bridged, maintaining the assumption of momentary behavior change resulting from contiguous reinforcement. If one is told in the morning to meet at the corner restaurant at 6 pm, the stimulus in the morning is said to change the response strength of the (still) latent response of going to the restaurant. Checking my watch at 5:45 pm provides an additional stimulus, which increases the response strength of the latent response of going to the restaurant until it becomes overt (Palmer, personal communication, June 13, 2016). This example suggests that response strength changes, especially when warranted by neurobiological events, represent a hypothesis about a mechanism at work in the organism, bridging the time elapsing between stimulus presentation and response emission.

## The Trouble with Palmer’s Conceptualization

### The Ontology of Response Strength: A Hypothetical Construct or an Intervening Variable?

Palmer (2009) explicitly calls response strength a hypothetical construct. MacCorquodale and Meehl (1948) have pointed to the importance of distinguishing “hypothetical constructs” from “intervening variables.” Failure to do so often leads to fundamental confusions. The two notions have been distinguished in many ways (e.g., Moore, 1995; Turner, 1965; Zuriff, 1985). For the present purpose, we adopt the distinction suggested by MacCorquodale and Meehl, which regards intervening variables as exhaustively defined, having no surplus meaning beyond the observations from which they are derived. Hypothetical constructs, in contrast, label possibly existing, hitherto unobserved entities or processes. They are not reducible to observed phenomena, in the sense that they carry surplus meaning beyond the observed events from which they are derived. As a reviewer of this manuscript noted, response strength might not satisfy the definition of a hypothetical construct when it is used as a descriptor of behavior, but response strength is often elevated in status from descriptor to explanation. Further, “response strength” as a descriptor has no apparent advantage over “probability” or “rate.”

Not all characteristics Palmer (2009) assigns to the notion of response strength point to a hypothetical construct. This ambiguity weakens what might be the closest things to a disambiguation of the concept of response strength adopted implicitly by most radical behaviorists. It leaves both the ontological status of the concept and the functional role “response strength” is supposed to hold in our explanations (Killeen & Hall, 2001) unclear. Is response strength something that we may eventually observe, such as the planet Neptune, which Adams and Leverrier (independently of each other) suggested to exist in an attempt to explain why the orbit of Uranus differed from what Newton predicted? Or is it something that we should not look for anywhere else but in an equation of other overt variables, such as the concept of resistance in electricity (i.e., “when we say that the resistance of a wire is such-and-such, we mean that so-and-so volts will give a current of so-and-so amperes"; MacCorquodale & Meehl, 1948, p. 2)?

In line with labeling response strength a *hypothetical construct*, Palmer (2009) regards rate, latency, force, resistance to extinction, prepotency over assumed competing responses, and EEG data as “indications” or “measurements” of the underlying factor of response strength. Reminiscent of, for example, personality or intelligence “measurements” in cognitive psychology, this “measurement” of a common hidden entity—not reducible to the measurements—supports the interpretation of surplus meaning.

Palmer (2009) writes that “It is important to note that response strength is a reflection of the status of the variables that control behavior and should *not* be viewed as *a property of the response* as an *independent* entity” (p. 59; emphasis added). If response strength is exclusively a function of the variables that control behavior, no surplus meaning is involved and no predictions of behavior based on anything but the observed controlling variables can be made. This resembles MacCorquodale and Meehl’s (1948) definition of intervening variables rather than hypothetical constructs.

Then again, Palmer also states on the same page:

It is true that sometimes such responses might be controlled entirely by the independent variables of which the putative behavior is a function, *but* in some cases it appears that they are controlled more directly by *a property of behavior that remains below the threshold of emission*. (p. 59; emphasis added)

This statement suggests that a per definition nonobservable response can contribute to the control of overt behavior, which is problematic in a scientific endeavor aiming to identify lawful relations between behavior and environmental factors. Palmer (2009) also asserts that “there is presumably an *important difference* between a response that is emitted and one that is not, regardless of their absolute levels of strength. Presumably *emitted responses have effects that can serve as controlling variables* for subsequent events” (p. 51; emphasis added). If latent events do not control subsequent events, the question arises as to what function they do serve in our analysis.

To be sure, neither hypothetical constructs nor intervening variables are in general problematic to explanations of behavior if used in a way that is logically consistent; however, there should be no ambiguity about ontological assumptions of the response strength concept. As Palmer (2009) is careful to point out:

Like all hypothetical constructs, a concept of response strength may invite reification and subsequent circular reasoning [and . . . he] would be more reluctant to invoke it if [he] were not persuaded that there are unifying physical instantiations of response strength that do indeed play a role in the control of behavior. (p. 59)

The danger of reification lies in reliance on explanatory fictions (Skinner, 1974) as causes of behavior. Explanatory fictions are characterized by circular reasoning occurring when the existence of an underlying factor is derived from an observation, which is then ascribed to that underlying factor. Curiosity tends to rest, and the actual functional relation between environmental variables and an organism’s behavior remains obscure (Baum, 2017).

Ranging from priming effects to problem-solving behavior, Palmer (2009) gives plenty of examples of behavioral change in which response strength change could be used as an explanatory factor. He also argues that changes in response strength of latent behavior may sometimes be “tacted” by “tip-of-the tongue”-phenomena. None of the examples includes a clarification of the reinforcement contingencies, and thus the relation between environmental factors and behavior remains unclear. The lack of the remaining parts of the explanatory chains in the examples given illustrates one of the risks one runs when including response strength as a part of an explanatory chain, even if not intending to regard response strength as *the* behavior initiating factor.

### Neurobiological Activity as Response Strength

Palmer (2009) explicitly states that “physical instantiations of response strength that do indeed play a role in the control of behavior” (p. 59) persuade him of the importance of response strength. He suggests changes in the N400 component of EEG recordings (e.g., Barnes-Holmes et al., 2005; Haimson, Wilkinson, Rosenquist, Ouimet, & McIlvane, 2009), which occur when words are repeatedly presented together, are instantiations of response strength. A neural change following a presentation of a discriminative stimulus in the absence of overt behavioral change may be interesting from a neurobiological standpoint. Yet it remains unclear how amplitudes of N400 waves support the proposed process of latent response competition, the winner of which increases in response strength until becoming observable. That is, brain activity does nothing above what behavior would do to make latent response competition measurable. Of course, to move, muscles require signals from the nervous system. Nevertheless, as Skinner (1953) pointed out, the immediate efficient cause of behavior is not inevitably the controlling cause. Trying to control behavior by eliciting one rather than another amplitude of the neural waveform occurring about 400 ms after the stimulus onset, is like trying to control a car by reaching inside and pulling the piston instead of moving the steering wheel, the accelerator, or the brakes (Rachlin, 2015). As the movements of the car are controlled at the border between the car and the environment (in this case the driver), behavior is controlled by manipulation of contingencies acting—if anywhere—at the border between the organism and the environment. The N400 is but one of many components of brain activity elicited in this case. If a series of stimuli lawfully precede a behavior change only when they are presented together, it appears trivial from a behavioral point of view that each stimulus changes some organismic variable. None of this is to say that the relation between brain and overt behavior is not important, but one must not forget that both brain and behavior require environmental inputs.

It would be risky to call the changes in N400 waves changes in covert responses or response strength for at least two reasons. First, the amplitude of the N400 varies with changes in how likely a stimulus is to occur in the context of the preceding stimuli (for a review, see Kutas & Federmeier, 2011). Although this does not preclude the N400 from indexing the strength of a covert response, the N400 might equally well index control by an extended pattern of environmental events in the absence of any change in strength. Indeed, brain activity that follows the delivery of a reinforcer for overt behavior also tends to be similarly influenced by the context in which those reinforcers occur—that is, by the sequence of events that typically unfolded in the organism’s learning history. The amplitude of various EEG frequencies is sensitive to contingency in a way that is not readily understandable as changes in response strength (McGill, Buckley, Elliffe, & Corballis, 2017). For example, McGill et al. (2017) showed that the same reinforcers evoke different amplitudes of activity depending on whether they occur in a context that also contains noncontingent reinforcers or one that also contains neutral stimuli. At an even more basic level, dopamine neurons involved in reinforcement learning (see Schultz, Dayan, & Montague, 1997) respond *more* to reinforcers that are unexpected in the context of recent experience than to those that are expected (e.g., Bayer & Glimcher, 2005; Hollerman & Schultz, 1998; Mirenowicz & Schultz, 1994; Schultz et al., 1997). At many levels of analysis, the brain activity that corresponds with measurable reinforcer-related changes in behavior is not easily (or usefully) explained as changes in response strength.

Second, referring to brain activity as a change in response strength is risky because the functional concept of the operant is not shared with any particular electrophysiological concept. One can readily conceive of behavioral regularities to which no physiological regularity corresponds and vice versa. If changes in the N400 waves corresponded to response strength changes, who would be doing the responding? The brain is not responding; it carries out the subbehavioral activity that makes the more molar concept of behavior possible. To be sure, there is empirical evidence that behavior–environment interactions select activities measured by EEG (e.g., Miltner, Larbig, & Braun, 1986; Sommer & Schweinberger, 1992), therefore perhaps EEG activity could be satisfactorily treated as behavior. Nevertheless, EEG activity would need to be activity of the whole organism, in the sense that such behavior is not explained by other activities of or in the same organism and are instead part of behavior–environment interactions. This is a theoretical premise that constrains the concept of behavior: behavior is what the whole organism does, not what the brain does using the whole organism (Bennett & Hacker, 2003). Furthermore, even if one is interested in efficient causes of behavior, causation is not unidirectional from brain to behavior but interactive from behavior-to-brain and the other way around (Schaal, 2003).

Even if we consider brain activity as behavior in the same sense as a lever press or key peck, the brain’s behavior appears not to give any more insight into response strength than nonbrain behavior. That is, regardless of one’s perspective on whether the activity of the brain is behavior of the whole organism, to regard brain activity as an indicator of response strength neither resolves the problem of observing latent response competition nor the problems caused by the ambiguous use of the term “response strength.”

We can be sure that the probability of a response’s emission has a physiological correlate at any moment we may measure it, and with sufficient instrumentation, we might at some point continuously identify neurobiological changes correlating with changes in discriminative stimuli. However, hypothetical constructs serve to aggregate correlated variables according to an underlying concept to reduce the dimensionality of the data. It might be questionable whether a response’s rate, latency, force, resistance to extinction, prepotency over assumed competing responses, and associated EEG data should all be aggregated under an underlying concept of response strength. Rather, it appears to be an empirical question to what extent rate, latency, force, and resistance to extinction can be predicted by EEG images. Neurobiological patterns should be treated as intervening variables with no surplus meaning.

Palmer’s bottleneck metaphor, illustrated in the lower right-hand corner of Figure 1, suggests a competition of latent responses. This competition appears to result from a conflation of ultimate and proximate explanations, in the sense that a mechanism, which can at best explain how behavior comes about, is described in a behavior-analytic vocabulary developed to answer the question why behavior occurs. Neurophysiology might outline the mechanisms someday, but will not be helped by hypothetical neural mechanisms, running the risk of instantiation. Instead, identification of electrophysiological mechanisms will require the study of function to know what such mechanisms are trying to explain. After all, how does one know there are semantic priming effects which correlate with distinctive N400 wave forms? The priming effect is determined by the participant’s overt response to the overt stimuli.

It is evident that a behavioral scientist attempting to support the usefulness of the concept of response strength by references to brain activity, as Palmer (2009) does, would not go as far as suggesting that behavior does not constitute or contribute in any way to what it means to have a mind (e.g., as Barrett, 2016, understands Berns, Brooks, & Spivak, 2012). Neither would a behavior analyst subscribe to mind–body dualism or claim that a scan of a motionless dog’s brain is more informative about the nature of its mind than any aspect of its world that involves physical activity (Berns et al., 2012).

Nevertheless, Palmer’s (2009) argument that considering response strength as a neurobiological phenomenon is necessary to explain complex human behavior such as problem solving implies that the brain is what matters, and what we need to speculate about, because it takes inputs, processes information, and computes outputs. Too much focus on brain activity as an explanation of behavior could suggest that problems are solved by the brain. However, problem solving requires the behaving body, including a brain, and the environmental structures that we use to augment, enhance, and support whatever internal processes operate to help getting us through the day. After all, beavers build dams (Kemp, Worthington, Langford, Tree, & Gaywood, 2012) and tuna can swim faster than their own physical capacities allow because they find natural water currents and then use their tails to generate vortices (Triantafyllou & Triantafyllou, 1995). We use post-its and computer files and we put the keys where we cannot overlook them when we leave the house. Our habits arrange the environment to simplifying what would otherwise be demanding tasks (Barrett, 2016; Kirsh, 1995). If we do not want to limit ourselves to identifying physiological mechanisms, an important part of what makes us perform the way we do may thus be found outside our heads, rather than within them. The alternative selectionist approach suggested below does not, as Mace (1977) formulates it, attempt to clarify “what’s inside your head, but what your head’s inside of” (the title of Mace 1977).

A clear distinction between mechanism and function might help to understand our argument. If a change in response strength is treated as a link in a chain of events, it is at best a description or umbrella term for the effect of preceding events. Thus, response strength is synonymous with response probability. If so, it is unnecessary to conjecture of changes of strength on a level of “latent behavior.” Even when changes in neurobiological activity were not used as *indicators* of response strength (leaving room for speculations of what other surplus meaning this notion might have) but were instead *identified* with response strength, one would still have to explain *why* such changes in neurobiological activity occur. Pointing to the physiological mechanisms at work between the presentation of two sequential stimuli and a response might at best shed light on an aspect of the question *how* the response is produced. The answer to *why* those two stimuli have an additive effect on behavior can only be found in phylogenetic and ontogenetic selection processes. Palmer (2009) mentions latent response competition as one of the indicators of response strength. It is unclear to what the metaphor of the bottleneck, in which the responses of a repertoire compete, translates. Do latent responses reside somewhere when not being emitted, and what causal factor—if not their response strength—makes for the emission of some but not of others?

From an evolutionary perspective, the process by which behavior becomes more likely to occur (i.e., operant selection), may be assumed to be naturally selected (Skinner, 1981; Simon & Hessen, 2019). In general, both natural and operant selection function to enhance an organism’s fitness. It seems reasonable to assume that reinforcers are effective due to their consequences on an organism’s fitness (Baum, 2012). Because the organism’s contact with its environment consists of its overt behavior, selection acts on, or at least through, overt behavior. Thus, potential changes in covert or latent behavior need to result from the overt behavior–environment interaction. Indeed, in Palmer’s (2009) paradigm, latent behavior arises from the reinforcement of overt behavior. As a matter of course, behavior changes go along with neurobiological activity, but terming this activity “behavior” only invites confusion. Sometimes a discriminative stimulus is presented and overt behavior does not occur. In Palmer’s view (personal communication July 2, 2016), there is a momentary behavioral difference between the presentation of a neutral stimulus and the presentation of a discriminative stimulus, even if no change in overt behavior is observable. In our view, the discriminative stimulus may or may not induce a certain change in neurobiological activity; to speak of *behavioral change*, the stimulus must change extended overt behavior, either immediately or after some delay. To identify the relation between a neurobiological activity pattern and overt behavior requires an identification of overt behavior in the first place.

## An Alternative

Response strength as an explanation of behavior, as exemplified by Palmer’s (2009) conceptualization, has led to more problems than benefits for a science of behavior. Response strength cannot be measured directly; depending on how the term is used, response strength as an explanation is either a hypothetical or intervening variable. If used simply as a description that equates to probability, rate, or some other measure of behavior, it is superfluous and invites conceptual confusion. Two problems arise: first, response-strength–based explanations like Palmer’s tend toward the unobservable—in Palmer’s case, competition among latent responses to leave a bottleneck. Second, a reliance on response strength pushes explanations toward physiological events, potentially distracting the field’s focus on understanding the relation between behavioral outputs and environmental inputs. We consider here whether an approach that is not based on response strength can overcome these weaknesses associated with a response-strength account of behavior.

Reliance on response strength stems largely from a conceptualization of behavior as a series of discrete events. The approach we consider here—the molar approach (Baum, 2012; Baum, 2015; Baum & Davison, 2014; Hineline, 2001, 2011; Rachlin, 1999, 2000, 2003, 2007; Simon, 2020)—differs from Palmer’s (2009) because 1) it does not invoke any notion of response strength; and 2) it views behavior as extended activities in time, rather than as discrete units. The molar approach regards operant selection as resulting from a competition between activities for an organism’s time (Baum, 2015). The total time that an organism can spend behaving is limited. An organism’s activities correlate differently with adaptively relevant events (Baum, 2012). Thus, contingencies between those activities and adaptively important events determine how much of an organism’s time each activity takes up. There is no need for hypothetical constructs or intervening variables.

As reflected in Skinner’s (1969) concept of stimulus control, molar approaches emphasize that subsequent behavior is not merely determined by its consequences but also by the stimulus context. The stimulus properties of reinforcers function as signposts, guiding or inducing behavior (e.g., Baum, 2012; Baum & Davison, 2014; Cowie & Davison, 2016; Shahan, 2010). It is important to note that the molar approach does not invoke any notion of response strength—hence we consider it here as an alternative to Palmer’s approach. The molar approach builds on less unfalsifiable assumptions and is therefore more parsimonious than the problematic notion of strengthening discussed above. As particle physics provides little information about responses on fixed ratio schedules, the molar approach acknowledges that processes at higher levels of integration may act as units differently from the lower-level processes of which they are composed (Schaal, 2003). Here, we first explore the potential merit in viewing behavior as a temporally extended occurrence, and then we assess whether the molar approach overcomes the problems associated with a response-strength account—namely, the temptation to focus on the unobservable (latent bottlenecks) and/or physiological events (EEG instantiations of response strength) as the explanation for behavior.

### The Trouble with Discretizing Behavior

Hypothetical constructs like response strength appear necessary to an explanation of behavior in part because of a focus on behavior as a discrete event. We would typically treat a blink of the eye or a gunshot as discrete events, that is, events whose duration is regarded as insignificant compared to the time passing between those events. Most often, unobserved variables are included to bridge the ostensive time gaps between those events and are conjectured to map on physiological characteristics, frequently located in the brain. The concept of strength was necessary to bridge the gap between discrete responses and the measure actually studied—response rate. With the understanding that behavior is necessarily temporally extended, the need for a concept like strength disappears, along with all its problematic properties. There is no need to talk about strengthening of extended activities: they just increase and decrease (Baum, personal communication, June 12, 2019).

In Palmer’s example mentioned above, for instance, where one is told in the morning to meet at a restaurant at 6 pm, the concept of response strength is used to explain why the stimulus in the morning changes the response strength of the (still) latent response of going to the restaurant. The additional stimuli provided by checking my watch at 5:45 pm increase the response strength of the latent response of going to the restaurant until it becomes overt. This example suggests that response strength changes, especially when warranted by neurobiological events, bridges the time elapsing between stimulus presentation and response emission via a mechanism at work inside the organism. One might question whether a response-strength change following the first stimulus is a satisfactory explanation for why I check my watch at the right time (5:45 pm). Instead of turning to hypothetical events inside the organism, one might consider that in the course of our ontogeny, behavior has been selected to discriminate between different periods of time, and most of us have learned to place in our environment reminding stimuli that will lead to the appropriate response. Without the habit of checking our calendars or watches, many of us forget appointments or birthdays. If we have acquired the habit of placing stimuli appropriately, the likelihood of the response increases. In our upbringing, our behavior has contacted both social and nonsocial reinforcers and punishers contingent on placing reminding stimuli where and when we will need them. The concept of response strength would add nothing but confusion to such an explanation. How we acquired the habit in our history cannot be tested for ethical reasons, but hypotheses about per definition latent behavior cannot be tested in principle, because a measurement would make the behavior public. Neurobiological changes might be measured, but there is no need to relate them to the ambiguous concept of response strength.

No researcher denies that all behavior takes time, but few use apparatuses that allow for measurements of variables with temporal extension. Throughout their history behavior analysists have used key pecks and lever presses, which discretize rats’ and pigeons’ continuous flow of behavior into countable events forming the basis of response rates. Had Skinner focused more on wheel running then pecking disks and pressing levers to which microswitches where attached, a different tradition might have developed. Sometimes a research question might be answered most straightforwardly by an analysis of a response rate consisting of counts; however, all interaction between an organism’s behavior and the environment—may it be the behavior of the researcher—is based on temporally extended behavior because if we were to regard behavior as momentary, an epistemological problem would arise. An example taken from Baum (2002) illustrates this problem. Looking at our snapshot depicted in Figure 2, can we tell what the rat is doing? Is it exploring? Is it sniffing the wall? Is it pressing the lever? Is it doing all or none of those activities? If this capture is to be the only controlling stimulus occasioning an observer’s verbal response, we will find the observer to be unsure about what the rat is doing. If, however, more of an animated sequence of which Figure 2 only is a part, is watched, certainty about the rat’s activity will increase. If we see what the rat did before and what it will do next, our verbal behavior will come under the control of those temporally extended stimuli. We do not know if someone sitting in a room with music playing is listening, or deaf and daydreaming, unless we observe them long enough to see if they, for example, start moving according to the music’s rhythm, or if their behavior remains unaffected if the music suddenly stops. The longer we observe the person’s activity, the more certain we can be of the activity’s function. Our understanding of the function of someone’s behavior does not only guide our everyday reactions but is also a prerequisite to formally analyzing an activity’s interaction with the environment. Baum (1997) makes an analogy with Heisenberg’s uncertainty principle. To know a pattern of behavior means to observe behavior for a period, the duration of which depends on the pattern being investigated. Exactly how much temporal precision and we need, or how much averaging is appropriate, depends on the research question and the measurement process.

According to the molar view, to analyze behavior means to analyze a sample of a temporal extended activity. As Baum (1997, p. 49) states it, “We cannot decide what a creature is doing without an adequate sample of its movement.” That is, in order to observe behavior, we need to observe the ongoing activity of an organism in a context, and that necessarily takes time. Carving behavior into discrete units may help to make it more easily quantifiable, but discretizing is likely to detract from our ability as scientists to explain and predict the behavior’s occurrence. To be sure, the concept of response strengthening, which most radical behaviorists implicitly subscribe to and which Palmer (2009) explicates, is a consequence of discrete response measurement, but measurement of discrete responses is neither particular nor original to Palmer’s view.

A view of behavior in terms of discrete events has a direct impact on research. Considering behavior as a discrete variable, even if implicit, affects research questions. When facing time gaps between discrete events, they are sometimes filled with hypothetical constructs such as “private events” and “response strength.” These events are allegedly present in the “changed organism” in some kind of presentism that struggles with the extended nature of behavior. At best, these theories could lead us to an empirical agenda based on physiological variables. At worst, they become instances of dualism.

### Can a Strength-Free Framework Avoid the Unfalsifiable?

In a temporally extended framework, dispositions or response probabilities become actual instead of hypothetical. Imagine we examine two locomotives. Both are sitting still, but one goes 100,000 miles in a year, and the other goes 50,000 miles in a year. Standing in the barn where the locomotives are kept, looking at them at a particular moment in time, we cannot tell the difference. Would the mileage be latent? Both rates are real and the only thing that matters from a pragmatic point of view—let’s say, to answer to maintainer’s question about whether worn parts need to be replaced (Rachlin & Frankel, 2009). Of course, an analysis of the locomotive’s mechanics may reveal that a particular gear had not worn out and does not have to be replaced; this, however, does not tell us anything about the locomotive’s behavior at a moment. Instead, the working gear reveals what might correspond to an organism’s current morphological characteristics, which are a function of both its behavior over time and its physiology, not of a hypothetical construct. Or as Ryle (1949) puts it:

To possess a dispositional property is not to be in a particular state, or to undergo a particular change. . . . My being an habitual smoker does not entail that I am at this or that moment smoking; it is my permanent proneness to smoke when I am not eating, sleeping, lecturing or attending funerals, and have not quite recently been smoking. (p. 31)

Does that mean Ryle is not a smoker when he is asleep or that smoking is latent when lecturing? The assumption of momentary behavior inevitably drives one to hypothetical constructs with only tenuous relations to actual behavior. Understanding response strength as an intervening variable, synonymous with response probability, would avoid the risk of reification. Then, a response’s rate does not indicate its strength but instead determines it. Such conceptualization as an intervening variable, rather than a hypothetical construct, would make locating response strength on different “levels of behavior” superfluous. It could not be positioned anywhere except from in the function of preceding variables. Because we may classify responses by their function or their topography, observed differences in force or resistance to extinction might be regarded as dependent variables in their own right. After all, if one would like to understand why one frequently talks silently or seldom talks loudly, it is of little help to regard the loudness and rate of talk as resulting from a common strength of a talking response. Whether we jump into a conversation depends not only on whether we have a point to make, but also on social contingencies requiring that we wait our turn. In this example, talking or not is not explained by the response’s strength, but by the contingencies of both talking and competing responses with so-called reinforcers.

## Can a Strength-Free Approach Explain Behavior Independent of Physiology?

The molar approach is an alternative to the radical behaviorist’s reliance on contiguous momentary behavior and its phylogenizing. The molar view factors in the temporal extension of behavior and the environmental events inducing it. The molar view is in line with epistemological arguments for regarding the behavior of whole organisms as the subject matter of behavior analysis as a selectionist science. The molar approach assumes that many conceptual mistakes interwoven with the concept of response strength arise from discretizing behavior (Baum, 2002). In this view, priming and additive effects of multiple sources of control by stimuli presented either sequentially or simultaneously, are no more in need for an explanation based on physiological or hypothetical events than other examples of stimulus control over behavior. There are physiological measures (e.g., EEG) that are correlated with a history of priming versus nonpriming procedures and the subsequent probability of a response, but these findings are interesting in their own right, not because they are interpreted as indicators of an ambiguous concept such as response strength. At the same time, empirical observations of behavioral patterns arising in priming procedures are interesting and complete for us without reference to proposed covert or latent mechanisms.

In line with molar behaviorism (Baum, 1995), interbehaviorism (Kantor, 1923), teleological behaviorism (Rachlin, 1991), and at times Skinner’s views (see Rachlin, 1995, for a discussion of the topic in the light of Skinner's biographical development), we would like to develop a science of behavior based on the responses of whole organisms in the sense that behavior is not explained by other activities in the organism but as behavior–environment interactions*.* To be sure, parts of the organism participate, but separately they do not engage in psychological activity. Skinner (1938) criticized the use of the nervous system as an "explanatory principle." Although the nervous system obviously responds to environmental inputs, a science of behavior loses sight of its goals when it replaces “environment” with “nervous system.” Indeed, responses often take more time than physiological events, and discretizing responses can run the risk of carving a behavior into units too small to be meaningful.

## Conclusions

This article advocates that the concept of response strength is ambiguous and superfluous. Dividing a continuous behavior stream into discrete units may have been a useful approach when trying to map out the basics of the interaction between behavior and environment, but it has also brought about superfluous problems, such as how to bridge time gaps between stimuli and responses. Response strength bridges time that passes between stimuli and responses in terms of discrete events, but in doing so it creates other problems for a science of behavior. In line with behavior analysis’ identity as a selectionist science (Skinner, 1981) and the arguments presented above, we suggest widening the search for explanatory variables in time rather than in space.

We might leave the search for intermediate controlling factors inside the organism to neurologists and continue our outline of controlling variables by going further back in an organism’s history. Describing neurobiological patterns at work in between discriminative stimuli and responses, such as the N400 component, in behavior analytic terms (e.g., as “latent behavior,” “covert response competition,” or “response strength”) instead of staying with the neurobiological terminology, impedes clarity. For example, the terminology of “latent behavior” (Palmer, 2009) may easily invite questions such as how we can know that a reinforcer has reinforced a latent response without analyzing overt responses. Naming neurological events in this way is only necessary when our theory of behavior relies on unobservable constructs; understanding behavior as an ongoing stream of activity in time removes the need for such constructs, and avoids any confusion over whether the behavior of the brain is latent behavior of the organism.

To be sure, Palmer’s point that changes in the N400 component occur when discriminative stimuli are presented serially even before changes of behavior of the whole organism are observable, is well-taken. It remains unclear, however, what practical purpose it might serve to call those neurobiological changes “latent *behavior*.” If observable by instrumentation (and as such, not *latent*), they might make our picture of the mechanics of an activity of a whole organism more complete. For logical reasons that have been discussed at length elsewhere (e.g., Bennett & Hacker, 2003), we argue for ascribing “behavior” to whole living organisms only.

If identifying latent behavior with neurobiological activity, one may just as well call a spade a spade. Identifying latent behavior with hypothetical constructs with a variety of instantiation invites confusion and runs the risk of creating pseudo-explanations. Moreover, an identification of neurobiological patterns does not reveal why some (combinations of) discriminative stimuli lead to certain responses whereas others do not. Neither does an identification of neurobiological patterns help to predict or control behavior in any applied setting. We put forward that the activity of the organism as a whole, extended across time and space, provides the most plausible and elegant data on which to base both theories and treatments of behavior. This activity need not be explained with any reference to the concept of response strength.

### Author note

Special thanks to David C. Palmer and William M. Baum for constructive feedback leading to valuable improvements of earlier versions of this manuscript. We would also like to thank Henrique Pompermaier and Mitchell Fryling for their support.

## Footnotes

1 In this article, we speak of Baum’s approach as a molar view. Following a suggestion from Hineline (2001), Baum refers to this view as a molar *multiscale* view in his later writings. Because a discussion of the multiscale aspects of the position would distract from our arguments, we simply use the terminology of Baum’s earlier writings by calling it the *molar* view.
